# Systematic review and meta-analysis for the use of ultrasound versus radiology in diagnosing of pneumonia

**DOI:** 10.1186/s13089-017-0059-y

**Published:** 2017-02-27

**Authors:** Saeed Ali Alzahrani, Majid Abdulatief Al-Salamah, Wedad Hussain Al-Madani, Mahmoud A. Elbarbary

**Affiliations:** 1 King Saud bin Abdulaziz University for Health Sciences, Riyadh, Kingdom of Saudi Arabia; 2 Emergency Medicine, College of Public Health and Health Informatics, King Saud bin Abdulaziz University for Health Sciences, Riyadh, Kingdom of Saudi Arabia; 3 National & Gulf Center for Evidence Based Health Practice (NGCEBHP), King Saud bin Abdulaziz University for Health Sciences (KSAUHS), Riyadh, Kingdom of Saudi Arabia; 4 KSAUHS, Ministry of National Guard-Health Affairs, King Abdullah International Medical Research Center, Riyadh, Kingdom of Saudi Arabia

**Keywords:** Systematic review, Ultrasound, Pneumonia, Point of care, lung, interstitial syndrome, and diagnosis

## Abstract

**Background:**

Physicians are increasingly using point of care lung ultrasound (LUS) for diagnosing pneumonia, especially in critical situations as it represents relatively easy and immediately available tool. They also used it in many associated pathological conditions such as consolidation, pleural effusion, and interstitial syndrome with some reports of more accuracy than chest X-ray. This systematic review and meta-analysis are aimed to estimate the pooled diagnostic accuracy of ultrasound for the diagnosis of pneumonia versus the standard chest radiological imaging.

**Methods and main results:**

A systematic literature search was conducted for all published studies comparing the diagnostic accuracy of LUS against a reference Chest radiological exam (C X-ray or Chest computed Tomography CT scan), combined with clinical criteria for pneumonia in all age groups. Eligible studies were required to have a Chest X-ray and/or CT scan at the time of clinical evaluation. The authors extracted qualitative and quantitative information from eligible studies, and calculated pooled sensitivity and specificity and pooled positive/negative likelihood ratios (LR). Twenty studies containing 2513 subjects were included in this meta-analysis. The pooled estimates for lung ultrasound in the diagnosis of pneumonia were, respectively, as follows: Overall pooled sensitivity and specificity for diagnosis of pneumonia by lung ultrasound were 0.85 (0.84–0.87) and 0.93 (0.92–0.95), respectively. Overall pooled positive and negative LRs were 11.05 (3.76–32.50) and 0.08 (0.04–0.15), pooled diagnostic Odds ratio was 173.64 (38.79–777.35), and area under the pooled ROC (AUC for SROC) was 0.978.

**Conclusion:**

Point of care lung ultrasound is an accurate tool for the diagnosis of pneumonia. Considering being easy, readily availability, low cost, and free from radiological hazards, it can be considered as important diagnostic strategy in this condition.

## Background

Acute pneumonia or acute respiratory tract infection is considered the most common cause of mortality in children around the globe [1]. In adult, pneumonia also is a serious disease with increased rate of mortality and hospitalization [2, 3]. The diagnosis of pneumonia can be difficult and challenging in the emergency setting or in critically ill patients [4]. Many of the commonly used radiological signs are non-specific [5]. In daily practice, pneumonia diagnosis is based on clinical presentation through patient history and physical exam, plus radiological imaging commonly chest X-ray (and infrequently CT scan) that may help confirm the diagnosis particularly with equivocal clinical status. Early diagnosing of pneumonia is very important to promptly starting the treatment; otherwise, it can be life-threatening or associated with high morbidity particularly in critically ill patients who need immediate decision.

There are many diagnostic approaches to diagnose and evaluate pneumonia and every tool has its own diagnostic accuracy.

Flexible bronchoscopy or endotracheal aspiration usually is reserved for intubated patients. Blood samples, urinary antigens, and expectorate collections are among routine examinations that are performed once pneumonia is suspected. Collected specimens are sent to microbiology laboratories [6] which may take several days to have conclusive results. Bronchoscope can give useful information; however, it has its own limitations and contraindications such as patients with severe hypoxemia, recent myocardial infarctions, or significant cardiac arrhythmia. Being relatively invasive technique, it is also not possible to perform bronchoscope in all patients but only in selected cases [7].

Another diagnostic tool is computed tomography, which is considered as the gold standard in lung imaging in general. This tool is particularly useful in lung masses or cavitary abnormality and any changes in lung parenchyma either acute or chronic such as the cases of pneumonia, interstitial lung disease, emphysema, and malignancy. The limitations are several but most important are radiation hazards, cost, and logistics that limit its routine use. A major limitation is difficulty in transporting patients with critical conditions to imaging section which precludes markedly unstable patients either respiratory or hemodynamically [8, 9].

Nevertheless, chest radiography remains an important imaging tool that been used for long and still helping in diagnosing many abnormalities in the chest. Chest X-ray is considered as the most common diagnostic tool that has been used traditionally in daily practice for diagnosis of pneumonia, especially in critical conditions [10]. Many limitations in using portable chest X-ray have been well described and noticed such as quality of an X-ray film in addition to the risk of repetitive radiation exposure [11]. Some reports claim that removal of chest radiography from daily practice may not affect intensive care unit mortality [12].

Relatively recently, lung ultrasound was promoted as a modality that can overcome many of the above-mentioned limitations of other tools in the diagnosis of pneumonia in multiple settings [13]. Through the last 2 decade, the ultrasound has shown that it could play a major role in medicine and common practice in assessing the lung [14]. Traditionally, the accessibility of the lung by ultrasound was considered poor due to the air barrier. However, this position has been dramatically changed with tremendous amount of literature supporting the use of LUS in multiple conditions [15–17]. This diagnostic tool can be used easily and immediately as a bedside tool which give it a huge advantage [18]. Lung ultrasound was reported with high accuracy in many pathological lung conditions such as consolidation, pleural effusion, and interstitial syndrome compared to bedside chest radiography [19].

The aim of our study is to conduct systematic review (SR) followed by meta-analysis for the diagnostic power of lung ultrasound versus chest radiological imaging for the diagnosis of pneumonia in both adult and pediatric population through estimation of the pooled diagnostic accuracy measures.

## Methods

A systematic search of electronic databases was conducted, including MEDLINE, EMBASE, and Cochrane databases from 1990 to 2016 to identify the relevant articles in the effectiveness of ultrasound in the diagnosis of pneumonia. Hand search was then conducted on references of relevant studies. The search strategy followed Cochrane guidelines with using the terms “Ultrasonography, ultrasound, sonography, ultrasonographies, sonogram”; “pneumonia, Bronchopneumonia, Pleuropneumonia, severe Acute Respiratory Syndrome, pulmonary inflammation, bronchiolitis”; and “sensitivity or specificity” with its MeSH terms. No restriction for language or type of patients was made at the time of the search. We included in this systematic review all studies evaluating diagnostic accuracy of lung ultrasound as index test against chest radiological imaging (CXR or CT) as reference standard. We included in this SR patients with respiratory disease and symptoms of acute respiratory failure. The evaluation of pneumonia is a combination of clinical data, laboratory results, and chest imaging. In addition, articles that evaluated any sign of respiratory disease, symptoms, or acute respiratory failure were included. We included all types of patients’ pneumonia—both community- and hospital-acquired pneumonia—, children, adolescents, or adults. We have chosen to combine both adults and pediatric based on current literature suggesting that ultrasound findings in both are similar [17].

Two authors (SZ and WM) screened titles and abstracts for valid articles. Full-text articles were retrieved afterward. We developed an abstraction tables that includes year of publication, patients’ baseline characteristics, and diagnostic study data (numbers of true positive, false positive, false negative, and true negative test results). Disagreement in study selection and abstraction was resolved by discussion with the third reviewer (ME).

Two reviewers (ME and SZ) independently used the QUADAS-2 instrument to assess the quality assessment of the included studies [20]. This tool consists of key domains covering patient selection, index test, reference standard, flow of patients through the study, and timing of the index test(s) and reference standard. Each domain was assessed in terms of the risk of bias and the concerns regarding applicability.

Risk of bias was judged as “Low,” “High,” or “Unclear.” If all signaling questions for a domain are answered “Yes,” then risk of bias can be judged “Low.” If any signaling question is answered “No,” this flags the potential for bias.

The meta-analysis was conducted using Meta-Disc 1.4 [21]. Random effect model was used in all analyses. The diagnostic accuracy measures used in the analysis were sensitivity, specificity, and likelihood ratio for positive and negative test (LR+ and LR−). Heterogeneity was assessed using the I-squared statistic and Q test.

## Results

**Fig. 1:**
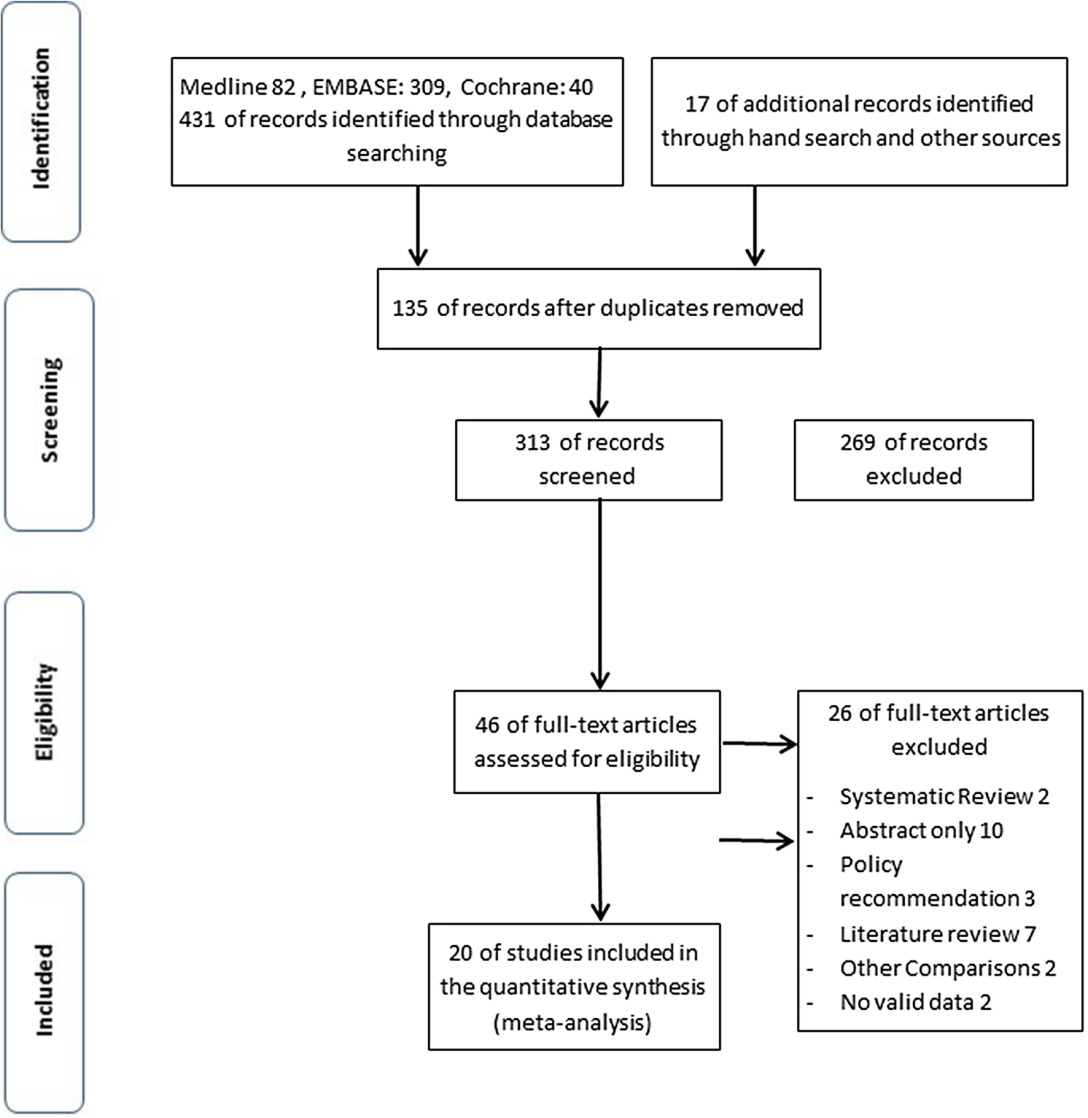
Flow chart for literature search process

We identified (431) studies that were relevant and fit our search strategy. After reviewing the articles and applying inclusion criteria and exclusion commentaries, we identified and enrolled 20 studies (see Fig. 1 flowchart). These 20 studies provided population of 2513 patients. The main reasons for exclusions were duplication of studies between the Pubmed and the Embase Databases and studies were not diagnostic.

Table 1 describes the basic characteristics of the 20 included studies. Among the included 20 studies, five of them are dealing with pediatrics patients [22–26]. Age of patients ranges from 1 month to 100 years. Some studies had comprehensive result of CT, clinical course, conventional tests, and follow-up outcomes as a diagnostic standard, which was considered clinical diagnosis. The quality of all studies was generally high, had low risk of bias, and satisfied the majority of the risk of bias criteria. Table 2 includes the chest imaging (reference standard) and other diagnostic criteria.

**Table 1:** Characteristics of studies and patients enrolled from studies retrieved for meta-analysis

Study	Year	Origin	Design	Sample size	Mean age (years)	M/F	True positive	False positive	False negative	True negative
Benci et al. [27]	1996	Italy	Prospective	75	38.5	50/30	37	0	0	20
Lichtenstein et al. [19]	2004	France	Prospective	32	58	Not mentioned	111	0	8	265
Lichtenstein et al. [28]	2004	France	Prospective	117	53	37/23	59	1	6	51
Lichtenstein et al. [29]	2008	France	Prospective	260	68	140/120	74	10	9	167
Parlamento et al. [30]	2009	Italy	Prospective	49	60.9	31/81	31	0	1	17
Cortellaro et al. [31]	2010	Italy	Prospective	120	69	77/43	80	2	1	37
Xirouchaki et al. [32]	2011	Greece	Prospective	42	57.1	34/8	66	4	0	14
Reissig et al. [33]	2012	Europe	Prospective	356	63.8	228/134	211	3	15	127
Testa et al. [34]	2012	Italy	Prospective	67	55	Not mentioned	32	5	2	28
Unluer et al. [24]	2013	China	Prospective	72	66.3	35/37	27	7	1	37
Luri [23]	2009	Italy	Prospective	32	4.5	60	20	2	8	2
Shah [35]	2012	US	Prospective	200	3	112/88	31	18	146	5
Hadeel and Dien [25]	2013	Egypt	Prospective	75	Neonates	Not mentioned	64	4	7	0
Copetti [36]	2008	Italy	Prospective	144	77.6	72/72	60	0	0	19
Caiulo VA [26]	2013	Italy	Prospective	88	5.1	56/47	88	0	1	13
Nafae [37]	2013	Egypt	Prospective	100	50.6	56/44	61	1	1	17
Liu [38]	2014	China	Prospective	179	72	124/99	80	0	5	27
Esposito [39]	2014	Italy	Prospective	103	5.6	56/47	52	3	1	47
Nazerian [40]	2015	Italy	Prospective	285	71.14	133/152	72	9	15	189
Bourcier [41]	2014	France	Prospective	144	77.6	72/72	117	9	6	12

**Table 2:** Chest imaging and diagnostic criteria of selected studies

Study	Imaging	Pneumonia diagnosis	Patient type	Inclusion criteria	Ultrasound operator	Diagnostic criteria	Blinding
Benci et al. [27]	CXR + Chest CT if CXR/LUS discordance	Clinical diagnosis or imaging	Hospitalized	Pneumonia symptoms	Experienced physicians	Consolidation	Yes
Lichtenstein et al. [19]	Chest CT	Imaging only	Critically ill	Acute respiratory distress syndrome	Experienced physicians	Consolidation	Yes
Lichtenstein et al. [28]	Chest CT	Imaging only	Critically ill	Chest pain or severe thoracic diseases	Two ED physician sonographers	Consolidation	Yes
Lichtenstein et al. [29]	CXR + Chest CT if possible	Clinical diagnosis or imaging	Critically ill	Acute respiratory failure	Experienced physicians	Alveolar and interstitial	Yes
Parlamento et al. [30]	CXR + Chest CT if CXR/LUS discordance	Imaging only	Presented to ED	CAP symptoms	Experienced physicians	Alveolar and interstitial	Yes
Cortellaro et al. [31]	CXR + Chest CT if possible	Clinical diagnosis or imaging	Presented to ED	CAP symptoms	Experienced physicians	Alveolar and interstitial	Yes
Xirouchaki et al. [32]	Chest CT scan	Imaging only	Critically ill	Mechanically ventilated patients scheduled for chest CT scan	Single physician (Expertise not mentioned)	Consolidation	Yes
Reissig et al. [33]	CXR + chest CT if CXR/LUS discordance	Clinical diagnosis or imaging	Presented to ED or hospitalized	CAP symptoms	Experienced physicians	Consolidation	Yes
Testa et al. [34]	CXR + chest CT if possible/indicated	Clinical diagnosis or imaging	Presented to ED	Suspected H1N1 infection	Experienced physicians	Alveolar and interstitial	Yes
Unluer et al. [24]	CXR + chest CT if possible/indicated	Imaging only	Presented to ED	CAP symptoms	Trained emergency physicians	Alveolar and interstitial	Yes
Nafae et al. [37]	Chest CT scan	Imaging only	Hospitalized	Pneumonia symptoms	Experienced physicians	Consolidation	No
Esposito et al. [39]	CR	Imaging only	Critically ill	CAP symptoms	Resident with limited experience	Alveolar and interstitial	Yes
Liu et al. [38]	CT scan	Imaging only	Presented to ED	CAP symptoms	Trained emergency physicians	Consolidation	Yes
Copetti et al. [36]	Electrocardiogram, Chest X-ray, and Color-Doppler echocardiography.	Imaging only	Critically ill	acute pulmonary edema	NA	Alveolar and interstitial	NA
Iuri [23]	Chest radiographs	Imaging only	admitted to the pediatric emergency ward	CAP symptoms	Two radiologists	Alveolar and interstitial	Yes
Shah [35]	Chest radiographs	Imaging only	patients had a routine clinical examination	Pneumonia symptoms	Trained physicians	Consolidation	Yes
Dien [25]	Chest radiographs	Imaging only	Critically ill	Pneumonia symptoms	One radiologist	Consolidation	NA
Caiulo [26]	Chest radiographs	Clinical diagnosis or imaging	Presented to ED	Pneumonia symptoms	One radiologist	Alveolar and interstitial	Yes
Nazerian [40]	Chest CT scan	Clinical diagnosis or imaging	Presented to ED	Any respiratory complaint	Trained emergency physicians	Consolidation	Yes
Bourcier [41]	Chest CT scan	Clinical diagnosis or imaging	Presented to ED	CAP pneumonia	Trained emergency physicians	Alveolar-interstitial syndrome	NA

**Fig. 2:**
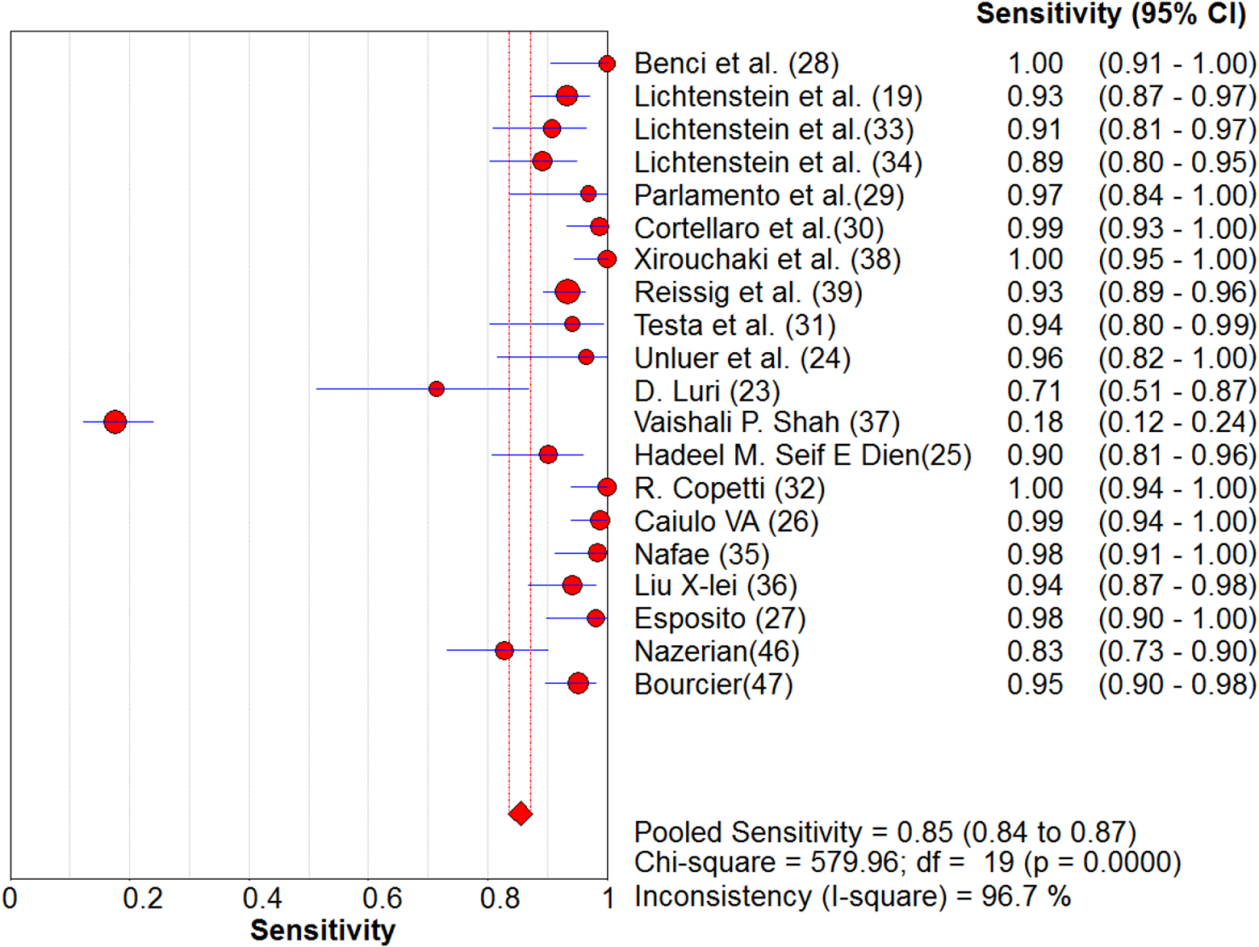
Pooled sensitivity of Ultrasound in ruling out pneumonia

**Fig. 3:**
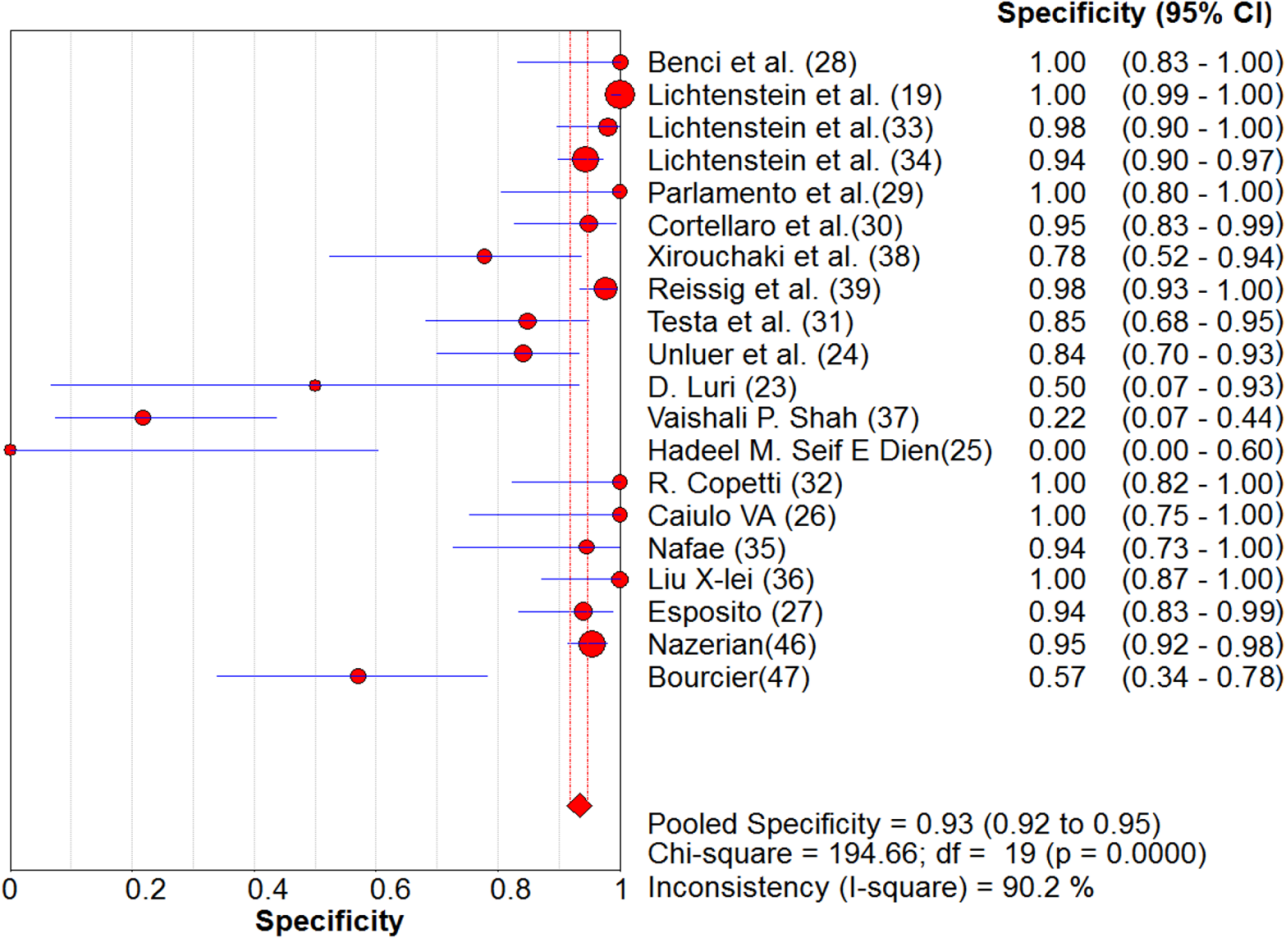
Pooled specificity of Ultrasound in ruling out pneumonia

**Fig. 4:**
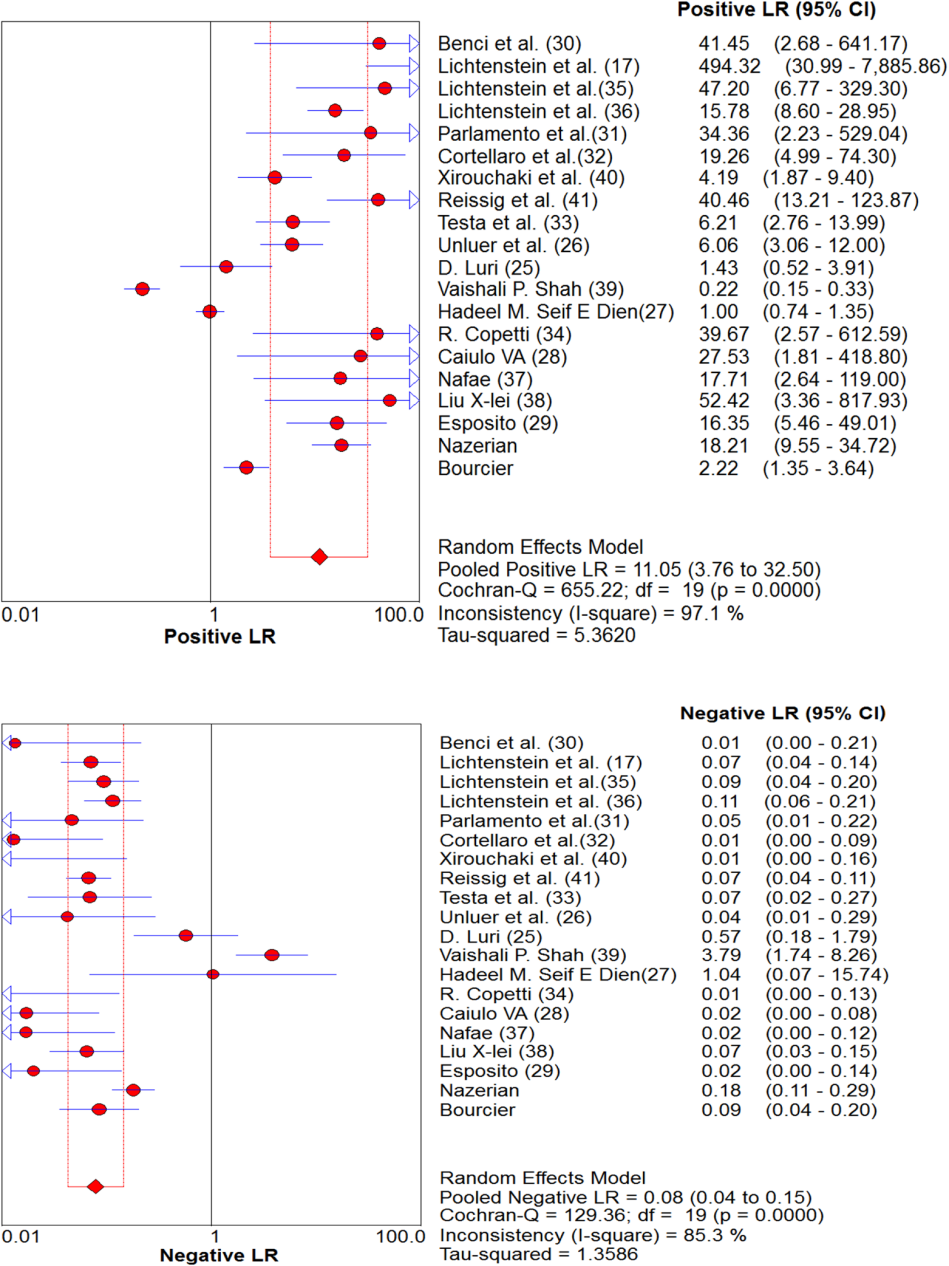
Pooled likelihood ratios of Ultrasound in diagnosing pneumonia

**Fig. 5:**
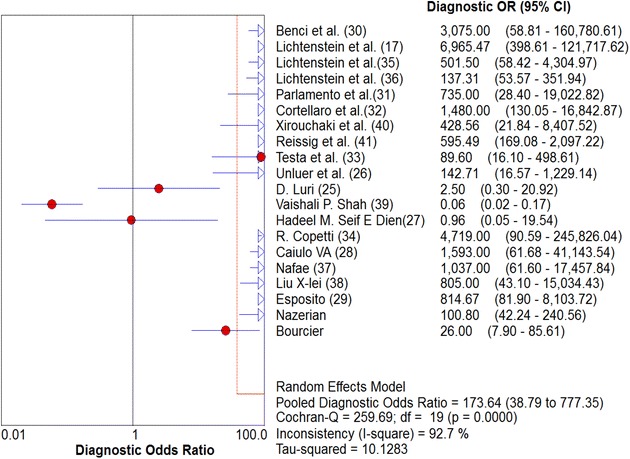
Pooled diagnostic Odds Ratio of Ultrasound in diagnosing pneumonia

**Fig. 6:**
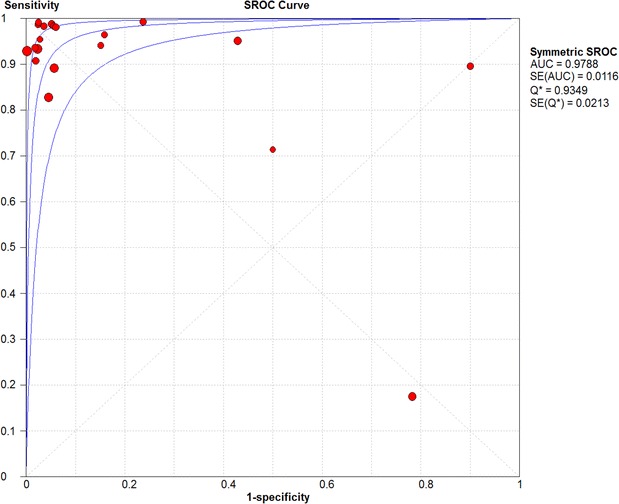
Pooled receiver operator characteristic curve of ultrasound in diagnosing pneumonia

Overall pooled sensitivity and specificity for diagnosis of pneumonia by lung ultrasound were 0.85 (0.84–0.87) and 0.93 (0.92–0.95), respectively (Figs. 2, 3). Overall pooled positive and negative LRs (Fig. 4) were 11.05 (3.76–32.50) and 0.08 (0.04–0.15), pooled diagnostic Odds ratio (Fig. 5) was 173.64 (38.79–777.35), and area under the pooled ROC (AUC for SROC) was 0.978 (Fig. 6).

## Discussion

Pneumonia commonly leads to significant pulmonary consolidation that is demonstrated with a complete loss of aeration in the concerned lung region. On CXR, pulmonary consolidation is defined as a homogeneous opacity that may have effacement of blood vessel shadows and the presence of air bronchograms.

In lung ultrasound, the normal lung displays the “lung sliding” and A-lines. Lung sliding indicates sliding of the visceral pleura against the parietal pleura and A-lines are repetitive horizontal reverberation artifacts parallel to the pleural line generated by normally present subpleural air in the alveoli.

On ultrasound examination, consolidation is defined as tissue-like pattern reminiscent of the liver, sometimes called “hepatization,” with boundaries that may be formed from the pleural line or a pleural effusion if present and the aerated lung, potentially forming an irregular scattered line if the consolidation is limited (shred sign) or a regular line if the whole lobe is involved. The LUS is logically capable in detecting superficial pneumonia, but it remains, however, doubtful in detecting deep alveolar lesions [39]. Consolidation is defined as an isoechoic tissue-like structure, which is caused by the loss of lung aeration. [4, 27] Power Doppler sometimes is used in order to differentiate tissue-like structures (e.g., echoic pleural effusion) from consolidation. The shred sign is specific for consolidation. B-lines are well-defined hyperechoic comet-tail artifacts, arising from pleural line and spreading vertically indefinitely, erasing A-lines and moving with the lung sliding when lung sliding is present. It indicates partial loss of lung aeration. Lung ultrasound using Doppler or contrast-enhanced sonography visualizes regional pulmonary blood flow within lung consolidations, thereby providing critical information about the etiology of the disease [27]. CXR does not provide any information about regional vascularization. The ultrasound detection of a dynamic air bronchogram is reported to be useful for differentiating obstructive atelectasis from pneumonia [27]. Several studies have demonstrated the superiority of lung ultrasound over CXR for diagnosing lung consolidation, particularly when portable CXR technique is used [30]. Therefore, the use of lung ultrasound can significantly reduce the number of chest radiographs and CT scans and decreases patients’ radiation exposure. It is easily repeatable at the bedside and provides more accurate diagnostic information than CXR in critically ill and emergency patients with lung consolidation.

In this study, we did a systematic review and meta-analysis for the diagnostic accuracy of radiological exam (CXR/CT) and lung ultrasound in relation to diagnosis of pneumonia. In comparison with previous systematic review published addressing this issue [4, 42], our study included more primary studies and subjects compared to previously published systematic reviews.

In our study, we found that lung ultrasound had a high LR, sensitivity, and specificity for the diagnosis of pneumonia. That represents a strong diagnostic accuracy measure with high precision as expressed by the relatively narrow 95% CI. It is important to emphasize that this high diagnostic accuracy can be operator-dependent [34]. The lung scan should be performed by well-trained operators in at least 6 zones to be able to achieve such high diagnostic accuracy [36]. However, in relation to CXR, previous 2 meta-analyses agrees about the superiority of ultrasound over portable CXR [4, 42].

This study emphasizes the role of lung ultrasound as an accurate technique for diagnosing pneumonia compared to chest radiological imaging. This comes in agreement with the multiple reports published for LUS use in multiple settings and new indication [43–47]. In addition, it can help in reducing the movement of patients to the radiology department for CT particularly in unstable mechanical ventilated patient.

## Limitation

Moderate-to-high degree of inconsistency/heterogeneity was observed which puts some caution for the interpretation of this study. The reason of heterogeneity can be due to differences in the population or in the reference standard (CXR and CT scan).

The study did not aim to investigate clinical end-point to prove/disprove LUS as a useful diagnostic strategy. That requires another SR of preferably RCT to elicit potential benefits of using the strategy of ultrasound diagnosis over radiological diagnosis. It will require examining several clinical outcomes such as earlier start of treatment, more effective management, reducing costs, reducing need for endoscope, and reducing complication such as cross-infection. These clinical endpoints were not addressed, as the focus was to establish pooled diagnostic accuracy rather than estimating effectiveness between comparative diagnostic strategies. However, our study managed to estimate high pooled diagnostic accuracy of this tool, which may justify its use.

In addition, we did not do comparison between LUS and chest X-ray in the general population (adults and children). That will require individual patient data (IPD) which are not available in the published studies. However, IPD meta-analysis has a robust methodology and peculiar characteristics that can be considered in this topic as potential future research.

## Conclusion

Lung ultrasound can play a major and valuable role in the diagnosis of pneumonia with high diagnostic accuracy. Moreover, it can be an alternative to chest X-ray and thoracic CT in several conditions. LUS can be used at the bedside easily, safely, and repetitively. Using LUS in Emergency department, ICUs, and medical wards after adequate training can be considered as a disruptive technology in this field.

## Abbreviations

LUS: lung ultrasound
CT: computerized axial tomography
X-Ray: X-radiation
LR: likelihood ratio
DF: degree of freedom
MeSH: medical subheadings
CXR: chest computerized axial tomography scan
QUADAS: quality assessment of primary diagnostic accuracy studies
